# Positive and Negative Effects of Administering a Magnetic Field to Patients with Rheumatoid Arthritis (RA)

**DOI:** 10.3390/jcm13061619

**Published:** 2024-03-12

**Authors:** Jolanta Zwolińska, Marta Kasprzak, Aleksandra Kielar, Michał Prokop

**Affiliations:** 1 Institute of Health Sciences, Medical College, University of Rzeszów, 35-959 Rzeszów, Poland; 2 Student Science Club for Investigation of Physical Energy Used in Physiotherapy, Institute of Health Sciences, College of Medical Sciences, University of Rzeszów, 35-959 Rzeszów, Poland

**Keywords:** rheumatoid arthritis, rheumatoid hand, magnetotherapy, static electromagnetic fields, pulsed electromagnetic fields

## Abstract

**Background**: Magnetotherapy applied to patients with rheumatoid arthritis (RA) produces anti-inflammatory, analgesic and antioedema effects. Observations suggest that the beneficial and adverse effects of magnetotherapy are related to the parameters of the magnetic field applied. This study aimed to assess the positive and negative effects of magnetotherapy, taking into account the type of the field. **Methods**: This study involved 39 patients with RA, who were randomly assigned to two groups: SMF—static magnetic field (*n* = 18) and PEMF—low-frequency pulsed electromagnetic field (*n* = 21). The examinations carried out before and after the therapy included a general assessment of the functional status, assessment of pain severity, measurement of the duration and severity of morning stiffness, computer-aided measurement of the range of motion of the hand joints and measurement of the hand volume using water displacement method. The patients received kinesiotherapy and magnetotherapy, as determined by the randomisation. **Results**: The findings show improved functional status by 0.26 points on average (*p* = 0.0166) measured with the Health Assessment Questionnaire (HAQ-20), reduced pain by 2.2 points on average (*p* = 0.0000) on the Visual Analogue Scale (VAS), decreased duration of morning stiffness by 23.2 min on average (*p* = 0.0010) and reduced severity of morning stiffness by 15.2 points on average (*p* = 0.0010). The assessment of the dominant hand showed improved range of motion by 1.9 mm on average (*p* = 0.0036) and reduced volume by 0.9 mm^3^ on average (*p* = 0.0230). A significantly reduced duration and severity of morning stiffness was observed in the SMF group. Statistically significant changes in the HAQ-20 scores, range of motion and the volume of the dominant hand were identified in the PEMF group. **Conclusions**: Magnetic fields improved the functional status and reduced pain, morning stiffness and swelling in the hand. A static magnetic field may be more effective in reducing morning stiffness, whereas a pulsed magnetic field may, to a greater extent, improve function and reduce swelling in the rheumatoid hand. The effects of magnetotherapy reported so far require further observation.

## 1. Introduction

The mechanism underlying the action of magnetic fields (MFs) on living organisms is extremely complex and, despite the continued scientific research related to this, the phenomenon still has not been fully explained [1,2,3]. MFs may be classified as static magnetic fields (SMFs) and those changing in time, i.e., dynamic magnetic fields [4]. Various categories of dynamic magnetic fields are distinguished depending on parameters such as the intensity, frequency and practical use [5,6,7]. Magnetotherapy applies magnetic fields with flux densities between0.1mT and 30 mT. The magnetic field pulses can be rectangular, triangular, trapezoidal, sinusoidal and sawtoothed. The frequency of these pulses is below 100 Hz, usually in the range of 5 to 50 Hz [7,8]. Significantly higher magnetic field induction values are used for transcranial stimulation in patients with depression [9].

Magnetic fields are widely used in the treatment of musculoskeletal diseases, including rheumatoid arthritis (RA) [1]. The outcome of magnetotherapy depends on the various parameters applied, such as the magnetic field flux density and frequency, as well as the shape of the field and the duration of exposure and treatment [10,11,12].

An in vitro study that assessed the action of LF-PEMF in cell cultures showed an anti-inflammatory effect of this type of field on osteoarthritic processes in the joints [13].RA is a chronic inflammatory arthropathy classified among the autoimmune diseases and it is associated with inflammation of the synovial membrane, whose high innervation generates pain and swelling and leads to chronic inflammation that mostly affects small, symmetrical joints of the hands and feet. Progressive deformation of the hand joints and accompanying pain lead to the deterioration of fine motor skills, which, in turn, adversely affects the performance of activities of daily living and significantly reduces the patients’ quality of life [4,14,15]. RA affects approximately 1% of the population worldwide and 0.66% of the population in Poland and is at least three times more common in women compared with men [16,17]. If it is not treated or correctly diagnosed, it can lead to irreversible changes, leading to disability [18]. In recent years, significant advances in the understanding of pathophysiological processes in RA made it possible to develop new management strategies [14]. One such may be feasible in patients with RA is magnetotherapy. Some studies showed that this method may be more effective compared with other physical factors [19,20]. Biological drugs and targeted therapies applied today are very helpful and improve patients’ quality of life [21,22,23,24]. Some patients, however, are not eligible for such treatment, or they do not respond well to these types of treatment and experience pain and impairments affecting the function of the upper limb [25].Our study proposed an alternative treatment method that can potentially be administered to patients who do not respond to biological treatments and other pharmacotherapy.This could also be an option for patients referred to rehabilitation treatments and are unable to systematically use outpatient physical therapy. In such cases, a practical solution would involve the self-administration of static magnetic fields emitted by permanent magnets to be used in home settings.

The treatment of RA requires an efficient long-term multidisciplinary approach [26]. Despite considerable progress in the related therapeutic methods, symptoms and dysfunctions experienced by patients in the rheumatoid hand continue to present a major problem for therapists. Pharmacotherapy applied in RA frequently leads to negative effects [27]. Valuable alternatives include physiotherapy involving motor exercise, as well as orthopaedic aids and massage. Beneficial effects are also produced by physical therapies, such as magnetotherapy, sonotherapy, laser therapy, cryotherapy and electrotherapy [28,29,30].

PEMF was shown to have anti-inflammatory effects by stimulating the differentiation of MSCs (mesenchymal stem cells/pericytes) into chondrocytes and osteocytes. Furthermore, PEMF increases collagen deposition and reduces vascular dysfunction while improving oxidation processes at the tissue level [19,31]. The use of magnetic fields to regulate inflammation and immune function is safer compared with other clinical immunosuppressive methods [32,33].

Magnetic fields are widely used in the clinical practice because of the anti-inflammatory, analgesic and antioedema effects produced by them. Moreover, magnetotherapy stimulates tissue regeneration processes and reduces muscle tension, which contributes to the patient’s improved functional status; it also seems to regulate blood pressure [1,6,8,12,27,28,34]. Magnetotherapy, being a non-thermal method, is safe and rarely causes negative effects [1,4,13]. The findings of many studies confirm the high effectiveness of magnetotherapy [35,36,37,38]. The anti-inflammatory effect and the stimulating impact on tissue regeneration were also shown by in vitro studies [36,39,40] and in research involving animals [3,41,42]. The results of preclinical research using animal models (mice, pigs, rats) show the positive effects of PEMF in musculoskeletal injuries and dysfunctions [3,41,42]. A study using murine material showed that PEMF can be used as an effective adjunctive therapy to inhibit the progression of RA [3]. It was also demonstrated that PEMF stimulates the improvement and recovery processes in rat tissue [41]. Furthermore, it slows down degenerative processes in the porcine articular cartilage. Conversely, some studies suggest that magnetic fields produce no effects [43,44].

Therapies generally apply an extremely low-frequency magnetic field (ELF-MF) and sometimes an SMF [10]. Even though magnetotherapy is widely used in clinical practice, the questions about the most effective type and parameters of the magnetic field are still valid. Consequently, there is a legitimate need to continue well-designed, high-quality research, which will provide findings that make it possible to standardise the treatment parameters and to develop optimal methods for use in contemporary healthcare [7,9]. It is also worthwhile to take into account the possible negative effects and safety issues related to magnetotherapy. Well-designed studies should also consider the possible adverse effects of magnetotherapy administered to individuals with RA. Safety issues related to the administration of magnetotherapy should also be taken into account. The authors hope that the article will fill a gap in the scientific literature related to this subject matter.

This study aimed to assess the positive and negative effects of magnetotherapy, taking into account the type of the field.

## 2. Methods

### 2.1. Ethics Approval

This study was conducted in compliance with the Declaration of Helsinki, and its protocol was approved by the Ethics Committee of the University of Rzeszów (resolution no. 2011/06/02). Participants gave written informed consent before the data collection began. The study protocol was registered onclinicaltrial.gov as NCT05920746. The day of first registration was 27 June 2023.

### 2.2. Study Design

This study was conducted and reported according to the CONSORT guidelines. All the data for this study were acquired through a double-blinded, 1:1 parallel-group, randomized trial. All the study participants were allocated to two parallel groups. A uniform kinesiotherapy program was applied in both groups. Additionally, static magnetic field therapy was applied in the SMF group, whereas the PEMF group received low-frequency pulsed electromagnetic field therapy. The patients were asked whether they experienced any negative effects after three and six therapeutic sessions.

### 2.3. Randomisation and Blinding

The patients referred for physiotherapy were randomly allocated into two groups. The randomisation was performed by members of the Student Science Club for Investigation of Physical Energy Used in Physiotherapy. The randomisation procedure involved tossing a1 zloty (1 PLN) coin. This method is commonly recognised as the simplest randomization procedure [45]. Heads meant allocation into the PEMF group and tails meant allocation into the SMF group. As a result of the procedure, 18 patients were allocated into the SMFgroup and 21 patients into the PEMF group. The study participants were not informed about which group they were assigned. Furthermore, the persons assessing the patients’ status (members of the Student Science Club who were not involved in this research) did not know the nature of the magnetic field applied in the therapy in this project.

### 2.4. Participants

This study was carried out in the Physiotherapy Laboratory at the Regional Clinical Hospital No. 2 in Rzeszów. Written information that detailed the purpose and course of this study was provided. The participants were also informed that they could withdraw at any stage of this study without stating their reasons. Seventy-two patients with RA referred for physiotherapy were invited to participate in this study. After taking into account the inclusion and exclusion criteria, 39 patients were ultimately enrolled for this study. The patients ranged in age between 36 and 80 years, and the mean age was 58.9 ± 12.9. They gave their written informed consent to participate.

1. The inclusion criteria were as follows:
  - Doctor’s referral for physiotherapy;
  - Any 2nd- and 3rd-degree radiological changes;
  - Any 2nd- and 3rd-degree functional changes;
  - Remission or low or moderate RA activity according to the DAS 28 index;
  - Voluntary, informed consent to take part in this study.
2. The exclusion criteria were as follows:
  - Contraindications to magnetotherapy, including unstable blood pressure;
  - Other physical treatments applied to hand area during the time of this study;
  - The use of steroidal anti-inflammatory drugs or strong analgesic drugs at the time of this study.

### 2.5. Intervention

The treatments were administered by members of the Student Science Club for Investigation of Physical Energy Used in Physiotherapy, who were supervised by a certified physiotherapist. Patients in both groups were subjected to a series of 10 physiotherapy procedures (kinesitherapy and magnetotherapy) performed on weekdays over three consecutive weeks. The interval between the treatments could not exceed three days. The maximum number of therapy sessions per week did not exceed five. The kinesiotherapy session was identical in both groups, lasting 30 min and included active exercise, a strengthening exercise with a tennis ball and manual resistance training using an exercise board. All the patients received magnetotherapy applied to the upper limbs (circular applicator with a diameter of 200 mm) over a course of 10 sessions, each with a duration of 20 min. The treatments were administered at the same time of day in all the patients. In line with the result of the randomisation procedure, patients in the SMF group were exposed to a static magnetic field (Magnetronic MF–10, manufactured by Elektronika i elektromedycyna, Otwock, Poland, File S1) with a flux density of 7 mT, and patients in the PEMF group were exposed to a low-frequency pulsed electromagnetic field (Magnetronic MF–12, manufactured by Elektronika i elektromedycyna, Otwock, Poland, File S2) with a flux density of 7 mT and a frequency in the range of 10 and 20 Hz with the use of rectangular bipolar impulse.

### 2.6. Outcome Measures

Before this study, all patients were examined by a specialist rheumatologist, who determined the degree of radiological changes, as well as functional changes and measured blood pressure [46,47]. Each patient was assessed for the Disease Activity Score in 28 Joints (DAS-28) [48].

The patients’ condition was assessed before the therapy (examination 1) and at the end of the intervention (examination 2).

The general functional status of all patients was evaluated using the Health Assessment Questionnaire (HAQ-20) [48]. It consists of 20 questions divided into eight sections. The patient assesses their functional status on a 4-point scale reflecting the difficulty experienced in performing a particular activity specified in the question, where “0”means no difficulty and “3” corresponds to complete inability to perform the activity [49]. The final score is the arithmetic mean of all the scores for the eight sections in the questionnaire.

The severity of pain was measured using the Visual Analogue Scale (VAS), with“0” meaning no pain and “10” reflecting the worst pain imaginable. In addition, an assessment of the hand was carried out, taking into account the following:

1. Duration of morning stiffness—as reported by the patient;
2. Severity of morning stiffness—a scale from 0 to 100 points was applied, with “0” meaning no morning stiffness and “100” reflecting maximum severity of morning stiffness [48].

Computer-aided measurement of the range of motion in the hand joints (the mean of three measurements) was carried our using an inductive sensor. This measurement is based on the principle of communicating vessels. The measuring cylinder, from which the medium is pumped, is connected to a flexible bellows, and the elongation of the bellows in the range from 0 to 45 mm makes it possible to transmit movement in the hand joints to the measuring sensor (Electronic Hand Assessment Set designed by Rzeszów University of Technology, Rzeszów, Poland) [50].

The hand volume was assessed using the displacement method [mm^3^], where the volume of water displaced was measured when the hand was submerged to the level of the distal edge of the ulnar styloid process (the mean of three measurements) [51].

### 2.7. Sample Size

The sample size was estimated based on the results of a pilot study and an analysis of the results obtained in similar research of this type, with a focus on hand volume, as this parameter is one of the key indicators in the practical assessment of rehabilitation effects (since it is an objective and precise measurement of hand swelling). It was assumed that the analysis would aim to detect, with a 90% power, the difference in effects of pulsed and continuous wave therapy at a level of 20 mm^3^. Additionally, it was assumed the estimated variability in rehabilitation effects (standard deviation) in each of the two groups would be at a level of 18 mm^3^. For these values, based on calculations for the independent samples t-test, the sample size in each group was obtained to be *n* = 19. The actual size of each group was *n* = 16, which was not far from the assumed level.

### 2.8. Statistical Analysis

The statistical analysis was carried out using non-parametric tests because of the deviations from the normality of the distribution in the case of a few performance measures and in order to harmonise the statistical methods used. The significance of the rehabilitation effects was assessed using the Wilcoxon test separately in SMF and PEMF groups. The significance of the differences between the groups was assessed using the Mann–Whitney test. The results of the tests are reported as *p*-values, and statistically significant values of *p* < 0.05, *p* < 0.01 and *p* < 0.001 are highlighted by the symbols *, ** and ***, respectively.

Data are available upon request from the corresponding author for the purpose of verifying the results in this study.

## 3. Results

### 3.1. Study Group

**Figure 1:**
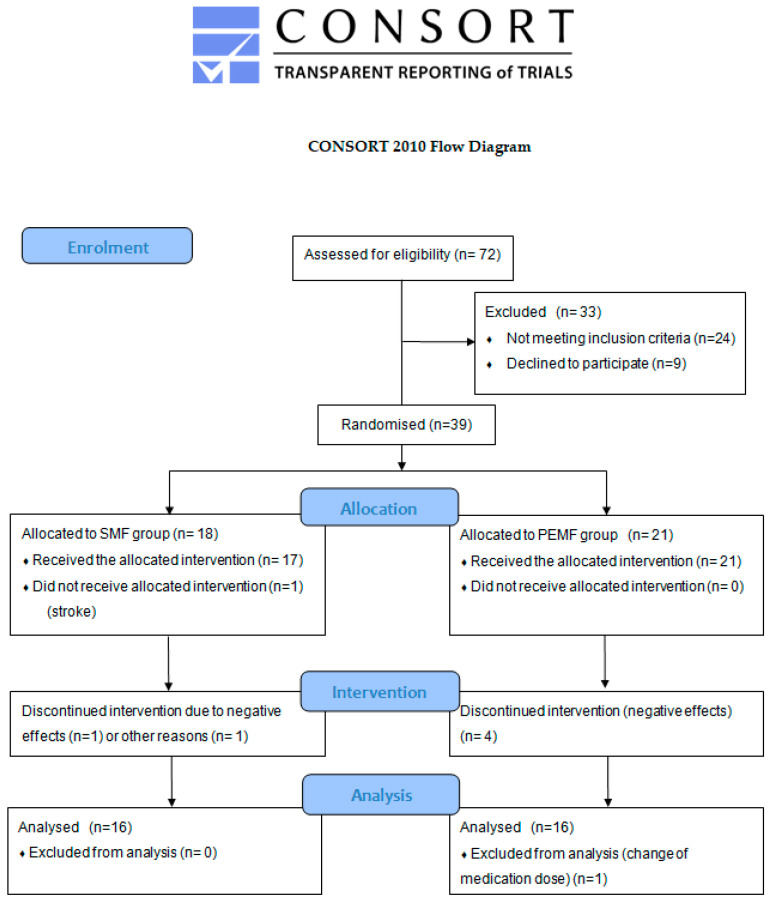
CONSORT flow diagram showing the progress of patients that received therapy through the phases of this study.

The initial stage of this study took into account 72 patients with diagnosed RA; in this group, 24 individuals did not meet the inclusion criteria and 9 refused to participate. The remaining 39 patients were randomly divided into two groups: 18 patients were assigned to the group treated with a static magnetic field (SMF group) and 21 to the group receiving low-frequency pulsed electromagnetic field therapy (PEMF group). Ultimately, this study took into account 32 participants. A detailed flow diagram is shown in Figure 1.

No differences were found between the two groups regarding the patients’ age, body mass index (BMI) and duration of the disease (Table 1).

There were no significant differences shown by the chi-squared test regarding the lateralisation (*p* = 0.5442) or advancement of the disease [degree of radiological changes (*p* = 0.5896) and degree of functional changes (*p* = 0.3770)].

### 3.2. Positive Effects of the Therapy

#### 3.2.1. General Effects

In the entire study group, the HAQ-20 test score decreased by an average of 0.26 points and this was a highly significant change. The probability value determined using the Wilcoxon test was *p* = 0.0166 *. Nearly identical improvement of the functional status measured with HAQ-20 was observed in both groups. The significant therapy effects measured using the HAQ-20 test were obtained only after the PEMF therapy. The probability value calculated using the Wilcoxon test was *p* = 0.0229 *. There were no significant differences between the groups regarding the effects of the therapy applied (Table 2).

The entire group showed a decrease in the intensity of pain, as assessed with the VAS, by 2.2 pointson average, and the change was highly significant. The probability value determined using the Wilcoxon test was *p* = 0.0000 ***. Both groups were found to have significantly decreased levels of pain. In the SMF group, the probability value determined using the Wilcoxon test was *p* = 0.0004 ***, and in the PEMF group, it amounted to *p* = 0.0058 **. The analgesic effect was more prominent in the SMF group; however, there were no significant differences between the two groups (Table 2).

The duration of morning stiffness in the entire group decreased on average by 23.2 min, and the change was highly significant. The probability value calculated using the Wilcoxon test was *p* = 0.0010 **. After the therapy, a shorter duration of morning stiffness was found in both groups; however, statistically significant improvement was only observed in the SMF group. The probability value determined using the Wilcoxon test in the latter group was *p* = 0.0051 **. In the PEMF group, the value was not statistically significant. There was no significant difference between the groups in the effect of the therapy applied (Table 3).

Changes in the severity of morning stiffness were identical for the entire study group. The severity of morning stiffness decreased by 15.2 points on average (on a scale from 0 to 100) and the change was highly significant. The probability value determined using the Wilcoxon test was *p* = 0.0010 **. Similarly, the severity of morning stiffness decreased significantly in the SMF group. The probability value calculated using the Wilcoxon test was *p* = 0.0080 **. In the PEMF group the effect only reflected a trend towards statistical significance (*p* = 0.0528). There was no significant difference between the groups in the effect of the therapy applied (Table 3).

#### 3.2.2. Therapy Effects in the Dominant Hand and the Subordinate Hand

The entire group showed an increase in the range of motion in the joints of the dominant hand by 1.9 mm on average, and the change was highly significant. The probability value determined using the Wilcoxon test was *p* = 0.0036 **. The range of motion in the joints of the dominant hand was improved in both the SMF group (by 0.7 mm on average) and in the PEMF group (by 2.9 mm on average). This change was statistically significant only in the PEMF group. The probability value calculated using the Wilcoxon test was *p* = 0.0125 *. There was no significant difference between the groups in the effect of the therapy applied (Table 4). The range of motion in the joints of the subordinate hand increased in the entire study group (by 0.5 mm on average). However, the change was not statistically significant. The range of motion in the subordinate hand decreased in the SMF group (by 1.1 mm on average) and increased in the PEMF group (by 2.0 mm on average). These changes were not significant statistically (Table 4).

Following the therapy, the volume of the dominant hand decreased in the entire study group (by 0.9 mm^3^ on average) and the change was statistically significant. The probability value determined using the Wilcoxon test was *p* = 0.0230 *. In the SMF group, there was a decrease in the hand volume (on average by 0.3 mm^3^); however, the change was not statistically significant. Conversely, in the PEMF group, the hand volume decreased by as much as 19.5 mm^3^ on average and the change was statistically significant. The probability value determined using the Wilcoxon test was *p* = 0.0038 ** (Table 4). Following the therapy, the entire study group showed a decrease in the volume of the subordinate hand (by 2.3 mm^3^ on average). The change was not statistically significant. In the SMF group, there was a decrease in the hand volume (by 8.1 mm^3^ on average); however, the change was not statistically significant. In the PEMF group, the hand volume decreased by 12.1 mm^3^ on average and the change was not statistically significant either. Nevertheless, the probability value determined using the Wilcoxon test reflected a trend towards significance and amounted to *p* = 0.0843 (Table 4).

**Figure 2:**
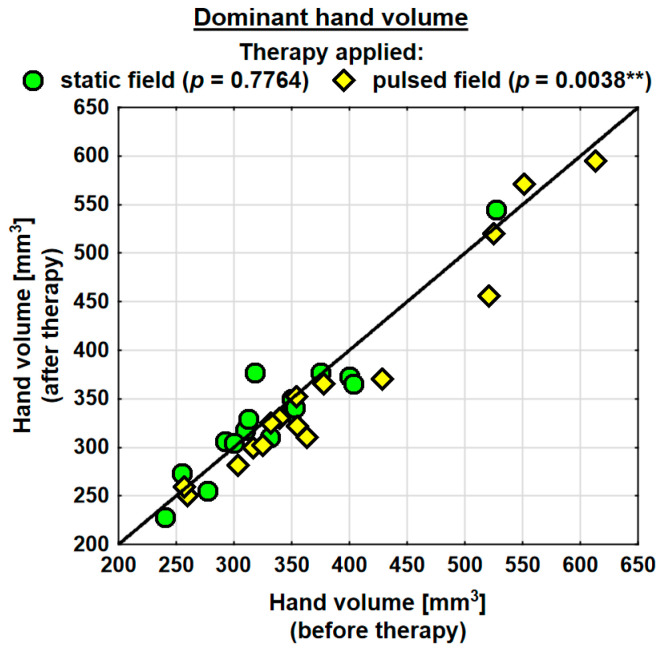
Changes in the volume of the dominant hand in the groups. Statistically significant values: **—*p* < 0.01.

**Figure 3:**
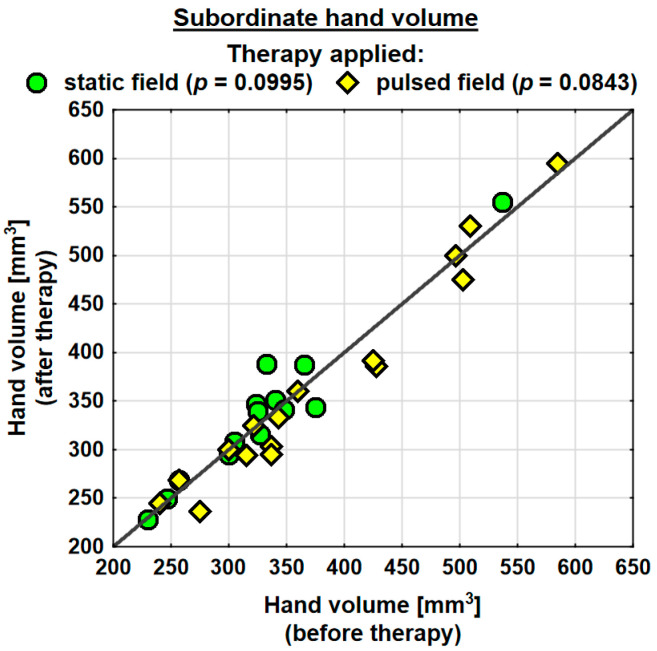
Changes in the volume of the subordinate hand in the groups.

Changes in the volumes of the dominant and subordinate hands in the specific cases are shown in Figure 2 and Figure 3.

**Figure 4:**
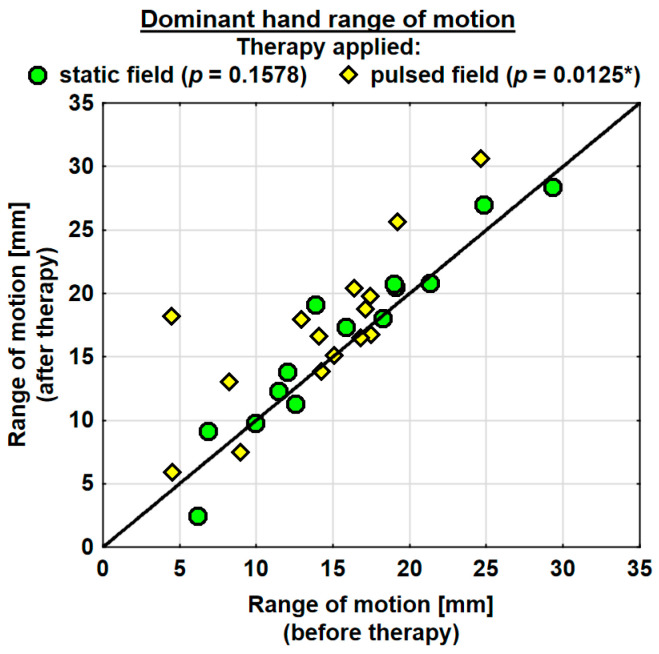
Changes in the range of motion of the dominant hand in the groups. Statistically significant values: *—*p* < 0.05.

**Figure 5:**
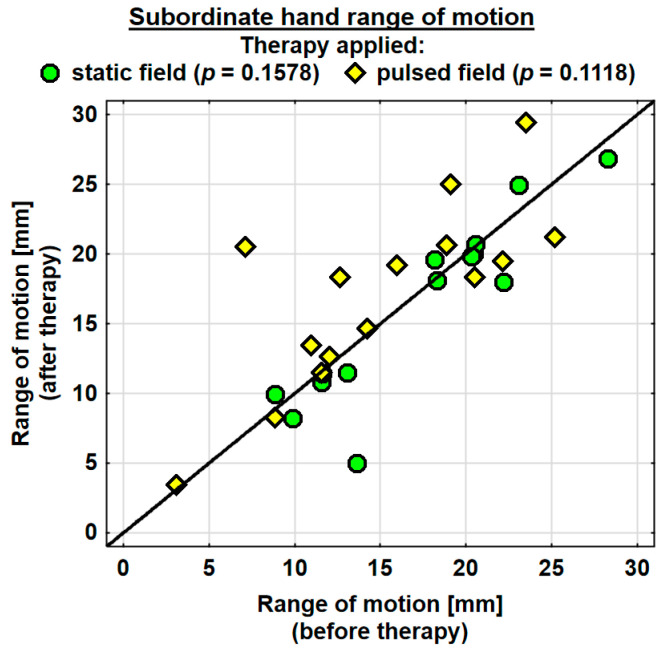
Changes in the range of motion of the subordinate hand in the groups.

Changes in the ranges of motion of the dominant and subordinate hands in the specific cases are shown in Figure 4 and Figure 5.

**Figure 6:**
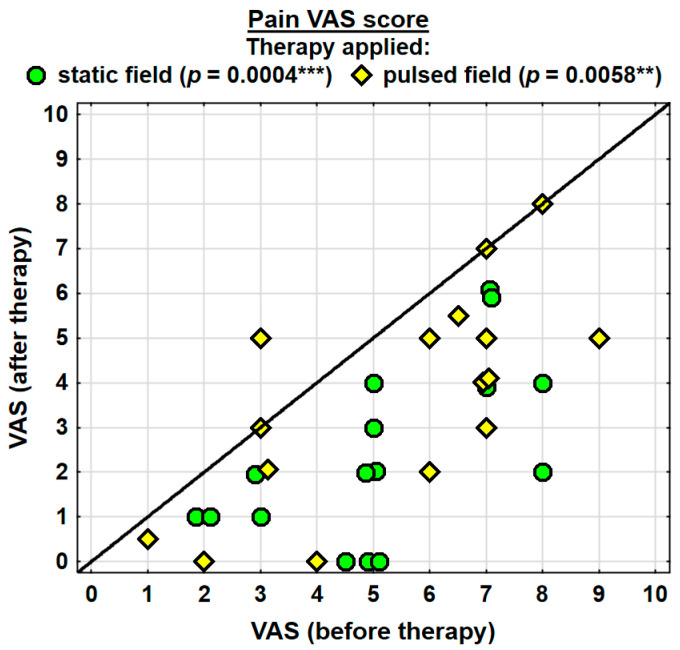
Changes in the pain VAS score. Statistically significant values: **—*p* < 0.01, ***—*p* < 0.001.

Changes in the pain (VAS) score in the specific cases are shown in Figure 6.

### 3.3. Negative Effects of Magnetotherapy

**Figure 7:**
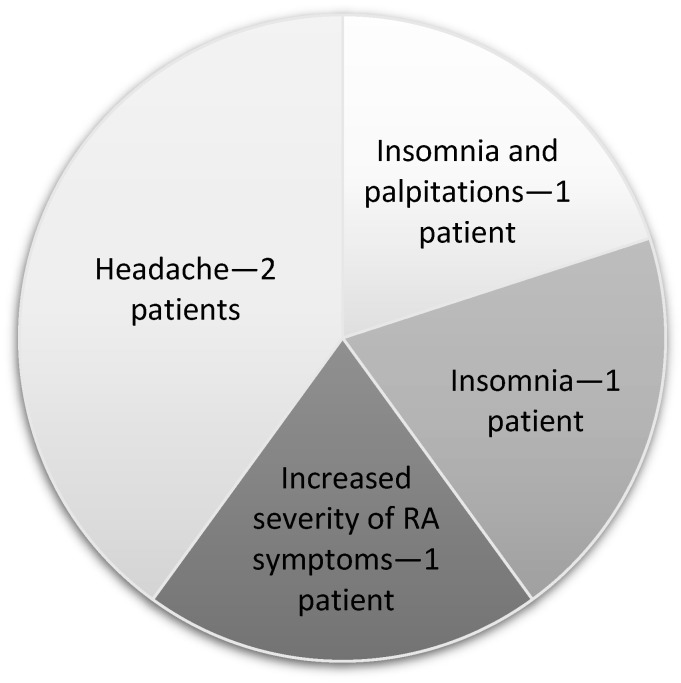
Negative effects.

In the entire group of patients that received the therapy, five individuals experienced adverse effects. These patients were not included in the final analyses. The adverse effects are shown in Figure 7.

## 4. Discussion

The effectiveness of various kinesiotherapy methods in patients with rheumatoid arthritis has been confirmed in a number of clinical studies [52]. In the case of acute and chronic pain associated with disorders of the musculoskeletal system, magnetotherapy is also applied as a safe and easy treatment method [34,53]. Many studies showed that the effectiveness of magnetotherapy is related to the nature of the magnetic field applied and the tissue sensitivity specific to the individual [2,3,34,53]. Many authors, however, emphasise the lack of precise research protocols, parameters of magnetic field applied and uniform assessment conditions [34,36,53,54,55].

The evidence reported by recent studies suggests that exogenous electromagnetic fields may be involved in many biological processes that are of great importance for therapeutic interventions [7,31,56]. Therefore, magnetotherapy has great potential to become a stand-alone treatment or adjunctive therapy for patients with musculoskeletal disorders. According to Tong et al. [56], it is still underestimated in clinical practice [7,57,58].

In the case of patients with RA, due to the variety and extensiveness of the symptoms experienced by them, choosing the most effective and safe physiotherapeutic methods is still problematic [26]. Due to their non-invasiveness and deep tissue penetration, magnetic fields are often used in therapy [12,19,53].

In the present study, the assessment carried out after the rehabilitation program, which consisted of hand mobility exercises and 20min magnetotherapy sessions, showed a significant improvement in the functional status in the entire study group evaluated using the HAQ-20 questionnaire, which is considered to be the most efficient method for assessing intervention outcomes in patients with RA [48]. However, statistically significant improvement in the HAQ-20 scores was only observed in the PEMF group. These results seem to be consistent with the findings reported by other authors, who observed that treatments with a duration of 30 min or shorter produce more beneficial effects compared with treatments with a longer duration [7,59]. Similarly, authors of a literature review reported that PEMF therapy improves the hand function in patients with degenerative joint disease [60].

The present study showed satisfying analgesic effects of the intervention in both the SMF and PEMF groups. Other studies also reported that the analgesic effectiveness of LF-PEMF therapies is higher compared with pharmacotherapy based on non-steroidal inflammatory drugs (NSAIDs) [61,62]. Furthermore, Shupak et al. showed that a single 30 min PEMF therapy session reduced pain in RA patients, although the authors expressed doubts about the durability of the effects achieved after a single exposure [63]. Similarly, in a study by Kalmus et al., the use of SMF reduced pain in patients with rheumatic diseases. Additionally, the findings showed an improvement in sleep quality and a reduction in inflammation in patients that received a spa treatment [64]. In contrast, Dündar et al. reported greater analgesic effectiveness of shortwave diathermy and electrotherapy treatments compared with PEMF [65].

The current findings also show a reduced duration of morning stiffness in SMF and PEMF groups. Likewise, a study by Kuliński and Skuza demonstrated that the duration of morning stiffness decreased from five to three hours in patients with stage 3 and stage 4 RA following a physical therapy intervention, which included magnetotherapy. This effect, however, was only sustained for four months [17]. On the other hand, the severity of morning stiffness in the current study was shown to decrease significantly in the SMF group. Stolarzewicz and Szczuka also compared the effects of static magnetic fields emitted by permanent magnets (SM) and low-frequency alternating magnetic fields (EM) on the severity of morning stiffness and found that the use of both EM and SM reduced the severity of the problem [66]. It also appears that LF-PEMF therapy is more effective in reducing the severity of morning stiffness compared with high-frequency PEMF [58].

The present findings also show that the ranges of motion in the joints of both the dominant and the subordinate hand were improved in the entire study group; however, the effect was better in the PEMF group. The lack of higher improvement in the range of motion in the subordinate hand in the entire group may have been linked to the fact that the subordinate hand was less engaged in the activities of daily living and self-care. A similar study was conducted by Stolarzewicz and Szczuka, who reported an improved range of motion in the knee following both SM and EM therapies [66].

In the current study, a significant reduction in hand volume was only observed in the PEMF group. In contrast, Chen et al. evaluated the distant effects of SMF application at a dose of 35 mT (magnetic knee wrap) and found no reduction in joint effusion [67].

Despite numerous studies confirming the beneficial effects of magnetic fields, there were some concerns about the safety of this therapy when it was being introduced [68]. Subsequent observations showed that magnetic fields with a flux density exceeding 10 mT can induce visual disturbances, such as flashes or shape deformations [69]. The literature reviews available in bibliographic databases show that no negative effects were reported in the participants of the studies conducted. In fact, the authors emphasised that magnetotherapy is well tolerated and can be a valuable adjunct to pharmacotherapy [4,60].

At present, researchers emphasise the occupational risks faced by physiotherapists associated with exposure to low-frequency magnetic field emissions during the treatments administered [59]. International standards issued by the World Health Organisation (WHO) and International Commission on Non-Ionizing Radiation Protection (ICNRP) permit environmental exposure to SMF with a flux density of less than 40 mT and less than 200 mT in the case of occupational exposure (8 h per day), except for individuals with electronic and ferromagnetic implants [70,71]. Safety of magnetotherapy procedures for both patients and the physiotherapists operating the magnetotherapy equipment require further study [9,59].

According to the WHO, in the case of electromagnetic hypersensitivity (EHS), patients may experience various symptoms, such as impaired concentration, sleep disturbances, excessive fatigue, dizziness, vomiting, palpitations and digestive disorders; non-specific dermatological symptoms: redness, tingling and burning; visual fatigue; and increased sensitivity to chemical stimuli [70,72]. Some researchers also mentioned the negative effects of SMF, such as headaches, nausea and vomiting, and skin lesions [73,74]. Furthermore, it was also suggested that an SMF may affect the course of neoplastic processes [75,76]. Some authors emphasise that an SMF with an extremely high flux density in some cases may cause adverse health effects [76]. An SMF with induction up to 8T was also found to adversely affect cardiovascular function; however, these effects were within the range of normal physiological variability. Furthermore, even when a flux density exceeding 2T was applied, some subjects reported dizziness and a metallic aftertaste in the mouth [77]. In fact, Driessen et al. argued that an SMF may produce negative effect when the value of the flux density is weak, up to one microtesla [55]. Both the WHO [70] and other authors [55] emphasise the methodological inadequacy and lack of precise magnetic field parameters in research investigating the exposure of the living organisms to SMF.

In a study by Thamsborg et al., negative effects occurred in both the PEMF-treated group and the sham magnetotherapy group. In that study, there were no serious adverse effects leading to discontinuation of the treatments. Mild and transient negative effects occurred during the first two weeks of treatment. Patients in both groups reported symptoms such as a grumbling or throbbing sensation, a warming sensation and aggravation of the osteoarthritic pain in the study knee [78]. According to this review, possible negative effects after PEMF therapy may include joint pain, vomiting, increased blood pressure, numbness of peripheral parts of the body and paraesthesia of the feet, as well as cardiomyopathy [60]. Since negative effects also occurred in the placebo-treated groups, the observations of the above authors [60,78] do not allow for a clear conclusion on the possible negative effects of magnetotherapy.

Other researchers emphasised that LF-PEMF therapy can lead to lower blood pressure and a slower heart rate [79]. In contrast, the authors of the review noticed the lack of assessment of negative effects in the studies discussed in the review [56]. Another important comment was contributed by Żurawski and Stryła, who noticed that the duration of time between exposure to LF-PEMF and the beneficial and adverse effects of the therapy are not strictly defined [80].

The participants of the current study had been affected by RA for 11.5 years on average and presented with stage 2 and 3 functional changes, as well as stage 2 and 3 radiological changes. According to Kuliński and Skuza, the effectiveness of rehabilitation is lower in patients with highly advanced RA [17]. Despite the fact that the patients presented with highly advanced RA, the current study found an improvement in all measured parameters. Nevertheless, the long-term effects of exposure to electromagnetic fields, both positive and negative, require further research [72].

## 5. Limitations and Strengths

A possible limitation of this study was the fact that the duration of the disease was greatly varied in the study population. The differences in the therapy effects between the SMF and PEMF groups may also be linked to the different baseline values of the parameters investigated (e.g., severity of morning stiffness and hand volume). For ethical and organisational reasons (lack of consent of most patients to participate in this study if the rehabilitation programme was limited to kinesitherapy only), the authors were not able to create a control group that was to be subjected only to kinesitherapy and not magnetotherapy. This study did not include a follow-up to assess the long-term effects. To enable this, the patients would have been required to give up any other forms of therapy necessary for RA for an extended period of time.In order to measure the long-term effects of the intervention, follow-ups at 3, 6 and 12 months would have been necessary.Despite these limitations, we need to emphasise that the eligibility criteria for participants were strictly defined, as were the rigorous conditions for the magnetotherapy. All the patients participated in a uniform kinesiotherapy programme. A general assessment of the patients’ condition (HAQ-20) was performed and a local, precise assessment of the hand was carried out.

## 6. Conclusions

Physiotherapeutic factors play an important role in the treatment of patients with musculoskeletal diseases. Magnetic fields improve function and reduce pain, morning stiffness and swelling. SMF therapy appears to be more effective in reducing the severity and duration of morning stiffness, whereas LF-PEMF therapy seems to more effectively improve function and reduce swelling in the rheumatoid hand. It is necessary to continue high-quality research on the negative effects of magnetotherapy, taking into account the long-term outcome of the treatments. It would also be important to assess the safety of both the patients receiving the treatments and the physiotherapists exposed to the magnetic field.

## Figures and Tables

**Table 1:** Study group characteristics.

	SMF Group (*n* = 16)		PEMF Group (*n* = 16)		*p*
	Mean ± Std. Dev.	Range	Mean ± Std. Dev.	Range	
Age [years]	58.9 ± 12.9	36–80	54.9 ± 13.7	30–77	0.4677
BMI	26.1 ± 4.4	19.8–37.7	27.6 ± 4.7	19.8–34.1	0.3809
Disease duration [years]	11.5 ± 9.5	0.5–30	14.4 ± 10.6	2–43	0.4016

**Table 2:** Changes in HAQ-20 scores and results of VAS in the two groups.

Examination	SMF Group		PEMF Group		Significance of Differences between Groups (*p*) ^(b)^
	Mean (95 c.i.)	Std. Dev.	Mean (95 c.i.)	Std. Dev.	
HAQ-20 Test					
Before therapy	1.58 (1.20; 1.96)	0.72	1.70 (1.30; 2.11)	0.76	0.5391
After therapy	1.30 (0.82; 1.79)	0.91	1.45 (1.07; 1.83)	0.71	0.5896
Therapy effect	−0.27 (−0.63; 0.08)	0.67	−0.25 (−0.53; 0.02)	0.52	0.5641
Significance of therapy effects (*p*) ^(a)^	0.1730		0.0229 *		
Pain VAS Score					
Before therapy	5.1 (4.1; 6.1)	1.9	5.4 (4.1; 6.7)	2.4	0.6420
After therapy	2.4 (1.3; 3.4)	2.0	3.7 (2.4; 4.9)	2.4	0.1188
Therapy effect	−2.7 (−3.6; −1.8)	1.7	−1.7 (−2.7; −0.7)	1.8	0.1381
Significance of therapy effects (*p*) ^(a)^	0.0004 ***		0.0058 **		

^(a)^ Result of Wilcoxon test. ^(b)^ Result of Mann–Whitney test. Statistically significant values: *—*p* < 0.05, **—*p* < 0.01, ***—*p* < 0.001.

**Table 3:** Changes in duration and severity of morning stiffness in the two groups.

Examination	SMF Group		PEMF Group		Significance of Differences between Groups (*p*) ^(b)^
	Mean (95 c.i.)	Std. Dev.	Mean (95 c.i.)	Std. Dev.	
Duration of Morning Stiffness [min]					
Before therapy	63.6 (36.3; 90.8)	51.1	114.1 (75.1; 153.0)	73.1	0.0513
After therapy	31.3 (12.4; 50.1)	35.4	100.0 (61.0; 139.0)	73.2	0.0022 **
Therapy effect	−32.3 (−53.5; −11.1)	39.9	−14.1 (−30.4; 2.3)	30.6	0.3226
Significance of therapy effects (*p*) ^(a)^	0.0051 **		0.0756		
Severity of Morning Stiffness					
Before therapy	45.0 (32.1; 57.9)	24.2	45.0 (34.4; 55.6)	19.9	0.9260
After therapy	26.3 (14.4; 38.1)	22.2	33.4 (21.1; 45.7)	23.1	0.4016
Therapy effect	−18.8 (−30.6; −6.9)	22.2	−11.6 (−23.0; −0.1)	21.4	0.4450
Significance of therapy effects (*p*) ^(a)^	0.0080 **		0.0528		

^(a)^ Result of Wilcoxon test. ^(b)^ Result of Mann–Whitney test. Statistically significant values: **—*p* < 0.01.

**Table 4:** Changes in the range of motion and hand volume in the two groups.

Examination	SMF Group		PEMF Group		Significance of Differences between Groups (*p*) ^(b)^
	Mean (95 c.i.)	Std. Dev.	Mean (95 c.i.)	Std. Dev.	
Range of Motion in Hand Joints [mm] (D) ^(c)^					
Before therapy	15.7 (11.9; 19.6)	6.7	14.1 (11.1; 17.2)	5.5	0.6516
After therapy	16.5 (12.4; 20.6)	7.1	17.1 (13.7; 20.5)	6.1	0.9829
Therapy effect	0.7 (−0.5; 2.0)	2.1	2.9 (0.8; 5.1)	3.9	0.1225
Significance of therapy effects (*p*) ^(a)^	0.1578		0.0125 *		
Range of Motion in Hand Joints [mm] (S) ^(d)^					
Before therapy	17.1 (13.8; 20.5)	5.8	15.0 (11.5; 18.6)	6.4	0.4773
After therapy	16.1 (12.3; 19.9)	6.6	17.1 (13.4; 20.7)	6.6	0.5613
Therapy effect	−1.1 (−2.6; 0.5)	2.6	2.0 (−0.4; 4.5)	4.4	0.0411 *
Significance of therapy effects (*p*) ^(a)^	0.1578		0.1118		
Hand Volume [mm^3^] (D) ^(c)^					
Before therapy	337.1 (300.4; 373.9)	69.0	388.9 (331.5; 446.2)	107.6	0.1381
After therapy	336.8 (299.3; 374.4)	70.5	369.4 (311.8; 426.9)	108.0	0.7520
Therapy effect	−0.3 (−12.8; 12.1)	23.4	−19.5 (−31.7; −7.3)	22.9	0.0615
Significance of therapy effects (*p*) ^(a)^	0.7764		0.0038 **		
Hand Volume [mm^3^] (S) ^(d)^					
Before therapy	327.0 (290.2; 363.8)	69.1	377.0 (322.5; 431.4)	102.3	0.2240
After therapy	337.2 (295.3; 379.1)	75.7	364.9 (307.8; 422.0)	107.2	0.8304
Therapy effect	8.1 (−2.8; 19.0)	19.7	−12.1 (−23.5; −0.6)	21.4	0.0215 *
Significance of therapy effects (*p*) ^(a)^	0.0995		0.0843		

^(a)^ Result of Wilcoxon test. ^(b)^ Result of Mann–Whitney test. ^(c)^ Dominant hand. ^(d)^ Subordinate hand. Statistically significant values: *—*p* < 0.05, **—*p* < 0.01.

## Data Availability

Dataset available on request from the authors.

## Supplementary Materials

The following supporting information can be downloaded at: https://www.mdpi.com/article/10.3390/jcm13061619/s1, File S1: MF-10 magnetotherapy apparatus with distribution of generated field force lines; File S2: MF-12 magnetotherapy apparatus with distribution of generated field force lines.
