# Supplementary material for: Positive and Negative Effects of Administering a Magnetic Field to Patients with Rheumatoid Arthritis (RA)
Source: J Clin Med. 2024 Mar 12;13(6):1619. doi: 10.3390/jcm13061619 (PMC10971695; doi:10.3390/jcm13061619)
Supplement: Supplementary file 1 [file jcm-13-01619-s001.zip › File S2.pdf]

OŚRODEK BADAŃ i ANALIZ „PP"
Marek Zając i Artur Zając s.c.
LABORATORIUM POLA ELEKTROMAGNETYCZNEGO
ul. Profesora Michała Bobrzyńskiego 23A/U2, 30-348 KRAKÓW
tel.: +48 603 57 77 88, +48 603 18 77 88, fax: +48 12 20 20 477
www.ppkrakow.pl, e-mail: artur@ppkrakow.pl, marek@ppkrakow.pl
ISTNIEJE OD 1989 R.

PCA
POLSKIE CENTRUM AKREDYTACJI
BADANIA
AB 286

Od 1 kwietnia 2000 r. posiadamy certyfikat akredytacji nr AB 286 wydany przez Polskie Centrum Akredytacji.

W ramach zakresu akredytacji wykonujemy:
- pomiary pola elektromagnetycznego (pole elektryczne, pole magnetyczne, gęstość mocy) w środowisku i w środowisku pracy w zakresie częstotliwości od 0 Hz do 90 GHz,
- pomiary hałasu w środowisku pracy,
- pomiary hałasu w budynkach mieszkalnych, zamieszkania zbiorowego i użyteczności publicznej,
- pomiary drgań:
  - o ogólnym działaniu na organizm człowieka,
  - działających na organizm człowieka przez kończyny górne,
- pomiary promieniowania optycznego nielaserowego, w ramach pomiaru przeprowadzamy dodatkowo pełną analizę skuteczności osłon na stanowisku,
- pomiary promieniowania laserowego,
- pomiary natężenia i równomierności oświetlenia na stanowisku pracy,
- pomiary oświetlenia ewakuacyjnego i awaryjnego,
- pobieranie prób powietrza w celu oceny narażenia zawodowego na: pyły przemysłowe (frakcja wdychalna + respirabilna).
- testy specjalistyczne medycznej aparatury rentgenodiagnostycznej w zakresie:
- radiografii ogólnej,
- stomatologii,
- mammografii,
- fluoroskopii i angiografii,
- tomografii komputerowej,
- monitorów do prezentacji obrazów medycznych.

Ponadto poza zakresem akredytacji wykonujemy:
- testy akceptacyjne medycznej aparatury rentgenodiagnostycznej,
- pomiary dozymetryczne osłon stałych,
- pomiary rozkładu mocy dawki wokół aparatów RTG,
- pomiary dawek referencyjnych w rentgenodiagnostyce,
- projekty pracowni RTG wraz z obliczaniem osłon stałych.,
- szkolenia z zakresu wykonywania testów podstawowych,
- opracowania dokumentacji Systemu Jakości w pracowniach RTG.

SPRAWOZDANIE

NR PP-PB/21-07-15-34

Z POMIARÓW PÓL ELEKTROMAGNETYCZNYCH WYKONANYCH W PRZESTRZENI PRACY W OTOCZENIU URZĄDZENIA DO MAGNETOTERAPII

1. MIEJSCE ZAINSTALOWANIA ŹRÓDŁA:
-województwo: **podkarpackie,**
-miejscowość: **RZESZÓW,**
-ul.: **Lwowska 60,**
-lokalizacja: **Regionalny Ośrodek Rehabilitacyjno-Edukacyjny Dzieci i Młodzieży - pracownia fizjoterapii - box 6.**

2. DANE DOTYCZĄCE ZLECENIODAWCY I UŻYTKOWNIKA:

-ZLECENIODAWCA: Kliniczny Szpital Wojewódzki nr 2 w Rzeszowie ul. Lwowska 60, Rzeszów.

-UŻYTKOWNIK: Kliniczny Szpital Wojewódzki nr 2 w Rzeszowie ul. Lwowska 60, Rzeszów.

-PRZEDSTAWICIEL ZLECENIODAWCY: Pani Marek Wojtasik.

3. POMIARY WYKONALI: mgr inż. Małgorzata Wyderska i mgr Aneta Bochenek.

4. DATA POMIARÓW: 19.07.2021 r., godz. $9^{00} \div 9^{30}$

5. OPRACOWANIE SPRAWOZDANIA Z POMIARÓW: mgr inż. Piotr Liniewicz.

6. DATA OPRACOWANIA SPRAWOZDANIA: 27.07.2021 r.

7. PRZEGLĄD WYNIKÓW i AUTORYZACJA: mgr inż. Artur Zając.

8. DATA AUTORYZACJI: 27.07.2021 r.

9. DANE TECHNICZNE DOTYCZĄCE ŹRÓDŁA PÓL ELEKTROMAGNETYCZNYCH:

Tabela 1.1 Informacje dotyczące źródła pierwotnego pola-EM.

nazwa urządzenia	Magnetronik
typ urządzenia	MF-12
producent	EIE Otwock
częstotliwość [Hz]	50
ilość włączonych aplikatorów	trzy cewki: 20cm, 30cm, 55cm,

Tabela 1.2. Parametry pracy źródła w trakcie pomiarów.

tryb pracy	ciągły
modulacja pola	sinus bipolarny
ilość podłączonych aplikatorów	3
indukcja	⌀20 - 11,2mT, ⌀30 - 5,6mT, ⌀55 - 2,8mT,

Dane zawarte w tabeli pochodzą z informacji uzyskanych od przedstawiciela Zleceniodawcy.
Wyniki pomiarów ważne są tylko dla badanego obiektu oraz pomieszczenia, w którym wykonywane były pomiary.

10. ZASTOSOWANIE ŹRÓDŁA – zabiegi medyczne.

11. CHARAKTERYSTYKA PRZESTRZENI PRACY I PRZESTRZENI OBSŁUGI.

11.1. W przestrzeni obsługi pracownicy użytkujący urządzenie:
-pracownik obsługujący badane urządzenie,
-serwisant urządzenia,
-montażysta urządzenia.

11.2. *Osoby potencjalnie narażone:*
-pracownicy wykonujący czynności związane z ochroną środowiska pracy (wykonujący pomiary związane z ochroną środowiska pracy).

12. DANE DOTYCZĄCE BADAŃ.

12.1. Cel badań: celem badań jest wyznaczenie zasięgu stref ochronnych wokół źródła pola-EM oraz określenie warunków narażenia na pole-EM w przestrzeni pracy.

12.2. W przestrzeni obsługi nie stwierdzono wtórnych źródeł pola-EM.

12.3. Czynności wykonywane są przez pracowników tylko przez czas niezbędny do ich wykonania. Przestrzeń obsługi pracowników użytkujących badane urządzenie w warunkach normalnej eksploatacji zlokalizowana jest w pobliżu źródeł pola elektromagnetycznego. Czas pracy pracownika zależy od wartości zmierzonego natężenia pola elektromagnetycznego w przestrzeni obsługi i wyliczany jest z odpowiednich zależności.

12.4. Warunki środowiskowe:

Tabela 2. Warunki środowiskowe.

Data	*godzina*	*pomiar*	*warunki wewnętrzne*			
19.07.2021r.	9:00	*początkowy*	*temperatura.:*	21°C	*wilgotność:*	49%
	9:30	*końcowy*	*temperatura.:*	22°C	*wilgotność:*	50%

12.5. Identyfikacja widma pola: identyfikacji źródeł i parametrów technicznych dokonano na podstawie analizy dokumentacji dotyczącej zlecenia oraz obserwacji miejsca wykonywania badań.

12.6. Aparatura pomiarowa.

Tabela 3. 1. Miernik natężenia promieniowania elektromagnetycznego.

1.	*miernik*		
	nazwa	miernik pola elektromagnetycznego	
	producent	Maschek Elektronik GmbH	
	typ	ESM-100	
	numer fabryczny	972605	
2.	*sonda pomiarowa*		
	typ	sonda zintegrowana z miernikiem	
	numer fabryczny		
	pole	elektryczne	magnetyczne
	zakres pomiaru pola elektromagnetycznego		
	dla f = 50 Hz	0,100 [kV/m] ÷ 50 [kV/m]	1,00 [μT] ÷19 [mT]
	zakres częstotliwościowy	5 [Hz] ÷ 600 000 [Hz]	10 [Hz] ÷ 600 000 [Hz]
	Niepewność zestawu pomiarowego	7,4%	11,3%
3.	*świadectwo wzorcowania*		
3.1.	*laboratorium wzorcujące*	Laboratorium Wzorców i Metrologii Pola Elektromagnetycznego (LWiMP)<br>Politechnika Wrocławska, ul. Janiszewskiego 9, 50-372 Wrocław; Nr akredytacji AP 078	
3.2.	*numer świadectwa wzorcowania*	LWiMP/W/344/20	

4.	*bieżąca kontrola sprawności zestawu pomiarowego*	zgodnie z aktualnie obowiązującą instrukcją sprawdzania zestawu pomiarowego.
5.	*świadectwo pomiaru odporności elektromagnetycznej*	
5.1.	*laboratorium wykonujące pomiar*	Laboratorium Wzorców i Metrologii Pola Elektromagnetycznego (LWiMP) Politechnika Wrocławska, ul. Janiszewskiego 9, 50-372 Wrocław; Nr akredytacji AP 078
5.2.	*numer świadectwa*	LWiMP/P/138/17
5.3.	*data wydania świadectwa*	21 grudnia 2017 r.

13. PODSTAWA METODYKI POMIARÓW.

13.1.Podstawy i Metody Oceny Środowiska Pracy 2016, nr 4 (90), s. 151 – 180.

13.2. Rozporządzenie Ministra Rodziny, Pracy i Polityki Społecznej z dnia 29 czerwca 2016 r. w sprawie bezpieczeństwa i higieny pracy przy pracach związanych z narażeniem na pole elektromagnetyczne, poz. 950 (tekst jednolity: Dz.U. z 2018 r. poz. 331).

14. WARUNKI PRACY OCENIONO NA PODSTAWIE:

14.1. Rozporządzenie Ministra Rodziny, Pracy i Polityki Społecznej z dnia 12 czerwca 2018 r. w sprawie najwyższych dopuszczalnych stężeń i natężeń czynników szkodliwych dla zdrowia w środowisku pracy (Dz. U. z 2018 r. poz. 1286).

14.2. Rozporządzenie Ministra Zdrowia z dnia 2 lutego 2011 r. w sprawie badań i pomiarów czynników szkodliwych dla zdrowia w środowisku pracy (Dz. U. z 2011 r. nr 33, poz. 166).

14.3. Rozporządzenie Ministra Rodziny, Pracy i Polityki Społecznej w sprawie bezpieczeństwa i higieny pracy przy pracach związanych z narażeniem na pole elektromagnetyczne (tekst jednolity: Dz. U. z 2018 r. poz. 331).

15. WYNIKI POMIARÓW.

Tabela 4. Wyniki pomiarów; wyznaczenie granic stref ochronnych.

numer pionu (punktu) pomiarowego	*opis miejsca pomiaru*	*wartość zmierzona natężenia pola magnetycznego po zaokrągleniu [A/m]*	*wysokość pionu (punktu) pomiarowego [m]*	*strefa*	*uwagi*
1	*2*	*6*	*7*	*8*	*9*
1÷14	-	266,7	1,0	**	Narażenie kontrolowane
15÷32	-	30,0	1,0	*	Narażenie kontrolowane

* - granica pomiędzy strefą bezpieczną a pośrednią,
** - granica pomiędzy strefą pośrednią a zagrożenia.

Tabela 5. Wyniki pomiarów; wartości natężenia pola-EM w przestrzeni obsługi, narażenie quasi-stacjonarne pola-EM.

numer pionu (punktu) pomiarowego	*opis miejsca pomiaru*	*wartość zmierzona natężenia pola magnetycznego po zaokrągleniu [A/m] na wysokości [m]*				*strefa*
		głowa 1,4 ÷ 1,8 m	*tułów 0,8 ÷1,2 m*	*kończyny*	M_{max}	
1	*2*	*8*	*9*	*10*	*11*	*12*
33	-	-	-	-	380,0/0,9	zagrożenia
34	-	-	-	-	200,0/0,8	pośrednia
35	-	-	-	-	58,0	pośrednia
36	Fizjoterapeuta	-	-	-	34,0/1,0	pośrednia
37	-	-	-	-	210,0/0,8	pośrednia
**Narażenie kończyn bezpośrednio przy powierzchni dostępu:**						
39	- Cewka 55cm	-	-	-	600,0	< IPNk-H [1]
40	- Cewka 20cm	-	-	-	1190,0	< IPNk-H [1]
41	- Cewka 30cm	-	-	-	740,0	< IPNk-H [1]
**Narażenie -Osoba pochylająca się :**						
Cewka 55cm						
	-Narażenie głowy (10cm prostopadle od obudowy)	310,0	-	-	-	zagrożenia
	-Narażenie tułowia 40cm prostopadle od obudowy)	-	<30	-	-	bezpieczna
Cewka 20cm						
	-Narażenie głowy (10cm prostopadle od obudowy)	310,0	-	-	-	zagrożenia
	-Narażenie tułowia 40cm prostopadle od obudowy)	-	<30	-	-	bezpieczna
Cewka 30cm						
	-Narażenie głowy (10cm prostopadle od obudowy)	520,0	-	-	-	zagrożenia
	-Narażenie tułowia 40cm prostopadle od obudowy)	-	<30	-	-	bezpieczna
**Narażenie - Osoba wyprostowana:**						
Cewka 55cm						
	-Narażenie głowy (80cm prostopadle od obudowy)	<30	-	-	-	bezpieczna
	-Narażenie tułowia - bezpośrednio przy obudowie aplikatora	-	600,0	-	-	zagrożenia
Cewka 20cm						
	-Narażenie głowy (60cm prostopadle od obudowy)	<30	-	-	-	bezpieczna
	-Narażenie tułowia - bezpośrednio przy obudowie aplikatora	-	1190,0	-	-	zagrożenia
Cewka 30cm						
	-Narażenie głowy (80cm prostopadle od obudowy)	<30	-	-	-	bezpieczna

16. ANALIZA WYNIKÓW ORAZ STWIERDZENIE ZGODNOŚCI.

16.1. Pomiary pola-EM wykonane w przestrzeni pracy w otoczeniu obiektu badań przeprowadzono w miejscach podanych w tabeli nr 4 i 5. Rozkład pionów (punktów) pomiarowych oraz położenie stref ochronnych przedstawiono załączniku nr 1.

16.2. Zgodnie z metodą wykonywania badań nie jest wymagane stwierdzenie zgodności. Miarą oceny narażenia na pole elektromagnetyczne jest określenie zasięgów stref ochronnych pola-E oraz pola-M. Zasięgi pola-EM stref ochronnych określane są w odniesieniu do limitów IPN zgodnie z wymaganiami określonymi w punkcie 14.1 sprawozdania.

16.3. Występowanie stref ochronnych.

16.3.1. W otoczeniu urządzenia do magnetoterapii będącego przedmiotem pomiarów **stwierdzono występowanie przestrzeni pola-EM strefy zagrożenia** (zgodnie z obowiązującymi przepisami dla strefy zagrożenia wartość **natężenia** pola-M wynosi powyżej 266,7 A/m do 16 000 A/m). Przebywanie w przestrzeni pola-EM strefy zagrożenia jest dopuszczane pod warunkiem stosowania środków ochronnych określonych ze względu na rozpoznane zagrożenia elektromagnetyczne wynikające z bezpośrednich lub pośrednich skutków oddziaływania pola-EM. Przebywanie w przestrzeni pola-EM strefy zagrożenia określane jest jako narażenie kontrolowane.

16.3.2. W otoczeniu urządzenia do magnetoterapii będącego przedmiotem pomiarów **stwierdzono występowanie przestrzeni pola-EM strefy pośredniej** (zgodnie z obowiązującymi przepisami dla strefy pośredniej wartość natężenia pola-M wynosi powyżej 30 A/m do 266,7 A/m). Przebywanie w przestrzeni pola-EM strefy pośredniej jest dopuszczane pod warunkiem stosowania środków ochronnych ze względu na rozpoznane zagrożenia elektromagnetyczne wynikające z pośrednich skutków oddziaływania pola-EM. Przebywanie w przestrzeni pola-EM strefy pośredniej określane jest jako narażenie kontrolowane.

16.3.3. **W miejscach poza strefami ochronnymi występuje przestrzeń pola-EM strefy bezpiecznej** (zgodnie z obowiązującymi przepisami dla strefy bezpiecznej wartość natężenia pola-M wynosi poniżej 30 A/m). Dla przestrzeni pola-EM strefy bezpiecznej nie określono warunków ograniczających ekspozycję. Przebywanie w przestrzeni pola-EM strefy bezpiecznej określane jest jako ekspozycja pomijalna.

16.4. Narażenie pracowników na pole-EM.

16.4.1. Dopuszczalny czas trwania ekspozycji w przestrzeni pola-EM strefy zagrożenia dla punktów pomiarowych w przestrzeni pracy podano w tabeli nr 5 (*cały punkt 16.4.1. poza zakresem akredytacji).*

Tabela 5. Dopuszczalny czas trwania ekspozycji w przestrzeni pola-EM strefy zagrożenia dla punktów pomiarowych w przestrzeni pracy.

nr punktu pomiarowego	*dopuszczalny czas pracy [h/dobę]*		*W*	*uwagi*
	[h/zmianę]	*[h, min/zmianę]*		
33	> 8,0 h	> 8 h 0 min.	< 0,01	czas narażenia - 30minut

16.4.2. Na podstawie przeprowadzonych pomiarów pola-EM w otoczeniu urządzenia do magnetoterapii będącego przedmiotem pomiarów **stwierdzono występowanie przestrzeni pola-EM strefy zagrożenia** w przestrzeni pracy.

Zgodnie z rozporządzeniem Ministra Zdrowia z dnia 2 lutego 2011 r. w sprawie badań i pomiarów czynników szkodliwych dla zdrowia w środowisku pracy (Dz. U. nr 33, poz. 166, § 11.1., pkt. 2.) pomiary należy przeprowadzać **co najmniej raz w roku** oraz każdorazowo, jeżeli nastąpiły zmiany w wyposażeniu technicznym, w procesie technologicznym lub w warunkach wykonywania pracy, które mogły mieć wpływ na zmianę poziomu emisji, poziomu narażenia albo wystąpiły okoliczności, które uzasadniają ich ponowne wykonanie.

Otrzymują:
1 x Zleceniodawca
1 x PP aa

Koniec sprawozdania. Sprawozdanie zawiera dodatkowo załącznik nr 1.

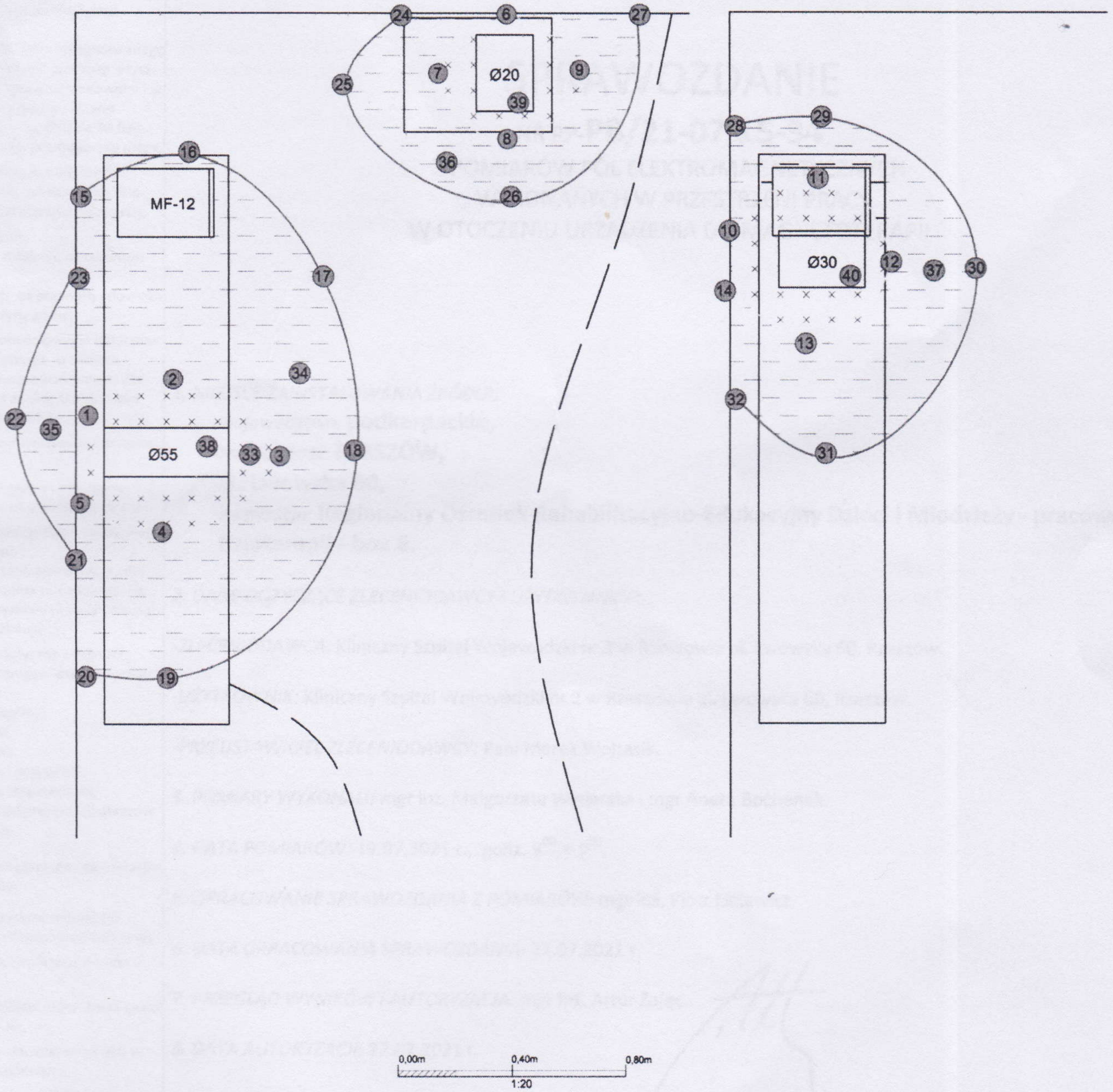

zał. nr 1:	Lokalizacja pionów (punktów) pomiarowych oraz stref ochronnych w Klinicznej Pracowni Fizjoterapii (Magnetronik MF-12).			
				-pion (punkt) pomiarowy.
strefy ochronne	-strefa	-strefa	- przestrzeń	

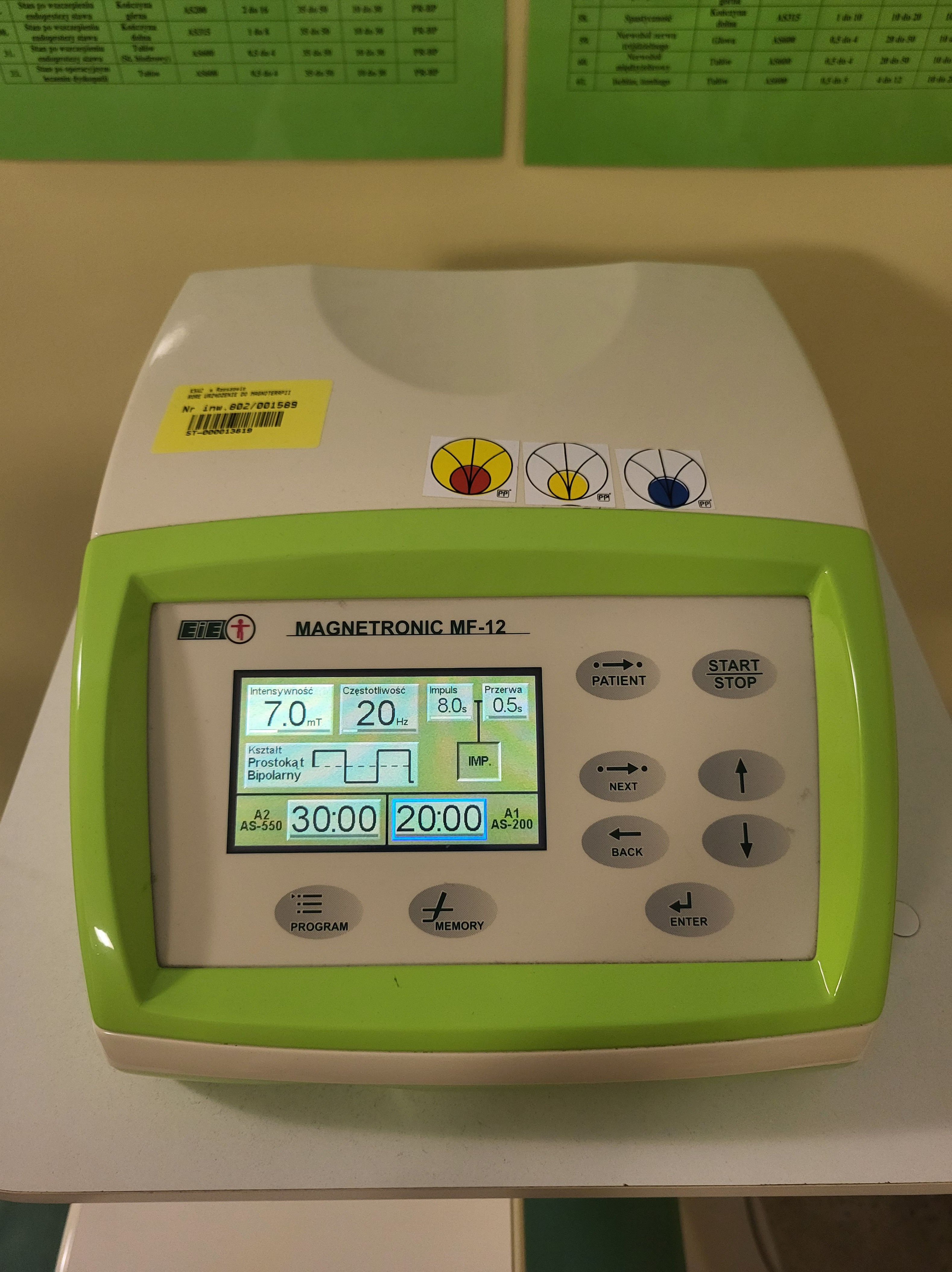
Nr inw.802/001589
ST-000013819
EiE
MAGNETRONIC MF-12
Intensywność
7.0 mT
Częstotliwość
20 Hz
Impuls
8.0 s
Przerwa
0.5 s
Kształt
Prostokąt
Bipolarny
IMP.
A2
AS-550
30:00
20:00
A1
AS-200
PATIENT
START
STOP
NEXT
BACK
PROGRAM
MEMORY
ENTER
